# Clinical and functional characterisation of the combined respiratory chain defect in two sisters due to autosomal recessive mutations in *MTFMT*

**DOI:** 10.1016/j.mito.2013.03.002

**Published:** 2013-11

**Authors:** Vivienne C.M. Neeve, Angela Pyle, Veronika Boczonadi, Aurora Gomez-Duran, Helen Griffin, Mauro Santibanez-Koref, Ulrike Gaiser, Peter Bauer, Andreas Tzschach, Patrick F. Chinnery, Rita Horvath

**Affiliations:** a Institute of Genetic Medicine, Newcastle University, Newcastle upon Tyne, UK; b University Children's Hospital, University of Tübingen, Germany; c Institute of Human Genetics, University of Tübingen, Germany

**Keywords:** Mitochondrial encephalomyopathy, Leigh syndrome, Mitochondrial translation, mt-tRNA modification

## Abstract

Exome sequencing identified compound heterozygous mutations in the recently discovered mitochondrial methionyl-tRNA formyltransferase (*MTFMT*) gene in two sisters with mild Leigh syndrome and combined respiratory chain deficiency. The mutations lead to undetectable levels of the MTFMT protein. Blue native polyacrylamide gel electrophoresis showed decreased complexes I and IV, and additional products stained with complex V antibodies, however the overall steady state level of mt-tRNA^Met^ was normal. Our data illustrate that exome sequencing is an excellent diagnostic tool, and its value in clinical medicine is enormous, however it can only be optimally exploited if combined with detailed phenotyping and functional studies.

## Highlights

• MTFMT mutations lead to mild Leigh syndrome and combined respiratory chain defect.
• Blue native PAGE showed decreased complex I and IV but normal mt-tRNAMet.
• Next generation sequencing (NGS) is a very powerful tool in mitochondrial disease.
• It can be optimally exploited if combined with phenotyping and functional studies.

## 1 Introduction

Mitochondrial protein synthesis is very complex, and the pathway requires ribosomal proteins, ribosomal assembly proteins, aminoacyl-tRNA synthetases, tRNA modifying enzymes, tRNA methylating enzymes and several initiation, elongation and termination factors of translation (Rötig, 2011; Smits et al., 2010). About 150 different proteins are involved in the translation of the 13 mitochondrial-encoded proteins, illustrating the importance of mitochondrial translation in human mitochondrial disease (Chrzanowska-Lightowlers et al., 2011; Rötig, 2011). Most of these gene defects result in cytochrome c oxidase (COX) deficient or ragged red fibres and biochemical multiple respiratory chain (RC) defect in affected organs. The clinical phenotypes are very variable, however most presentations are early-onset, severe, and often fatal, implying the importance of mitochondrial translation from birth (Kemp et al., 2011). Some of these conditions affect multiple tissues, however tissue specific manifestations have been reported for several mt-tRNA aminoacyl synthetase or mt-tRNA modifying genes (Chrzanowska-Lightowlers et al., 2011; Rötig, 2011).

Due to the rapid increase in the number of nuclear genes and the widely varying phenotypes associated with combined RC deficiency, it is extremely difficult to select specific candidate genes for diagnostic screening. As a consequence, sequencing the known nuclear genes affecting mitochondrial protein synthesis has not been efficient in an earlier study (Kemp et al., 2011). However, next generation sequencing (NGS) has proven to be a very powerful tool in identifying nuclear disease genes in combined RC deficiency (Calvo et al., 2010). Either analysing ~ 1000 genes predicted to encode mitochondrial proteins (MitoExome) (Calvo et al., 2010; Tucker et al., 2011) or studying the whole exome has successfully detected the causative mutations in several families with RC deficiency (Ghezzi et al., 2012; Haack et al., 2012; Shamseldin et al., 2012). Most of these papers report a low number of patients with a single gene defect however some genes appear across different cohorts, indicating the existence of some more frequent genetic defects (Hennekam and Biesecker, 2012). Although characteristic clinical presentations may facilitate the recognition of the phenotype in larger patient cohorts (Steenweg et al., 2012), this is not usually the case.

Mutations in the mitochondrial methionyl-tRNA formyltransferase (*MTFMT*) gene were first identified using targeted sequencing of the mitochondrial and nuclear encoded mitochondrial proteome (MitoExome) in two unrelated patients with Leigh syndrome and combined complex I deficiency and complex IV deficiency (Tucker et al., 2011). Exome sequencing in a cohort of patients with complex I deficiency identified two further patients with MTFMT deficiency, suggesting that this gene defect may account for a relevant number of patients with combined (I and IV) or isolated (complex I) deficiency (Table 1). Here we report clinical, biochemical, genetic and in vitro studies in two sisters carrying compound heterozygous mutations in *MTFMT*.

## 2 Materials and methods

### 2.1 Case reports

#### 2.1.1 Patient 1

Patient 1 was included in a cohort of 52 patients with genetically undefined combined respiratory chain deficiency (patient 12 in Kemp et al., 2011). She is currently 16 years old, the first child of healthy non-consanguineous German parents. She has a similarly affected younger sister and a brother, who has normal neurological status, but has behavioural problems. Birth and early motor development were normal (walking by 18 months of age), however speech development was delayed, leading to the first clinical investigations at 3 years of age (normal chromosome analysis and EEG). She slowly developed coordination problems over the following 3 years.

On clinical examination at 6 years of age her weight and height were < 3rd percentile. There was no ptosis or ophthalmoparesis. She had mild facial hypotonia, but normal vision and hearing. She had a slight dysarthria and speech was limited to short sentences. She had no muscle weakness, however muscle tone was generally reduced; deep tendon reflexes were normal and symmetric. There was a mild ataxia, causing difficulties in tandem gait, and her fine finger movements were clumsy. She could walk and run without help, could not jump, but learned to ride a tricycle. Her cognitive function was slightly impaired.

Cardiac and respiratory functions were normal. Laboratory tests were normal, except for mildly increased CSF lactate (3.3 mmol/L, normal < 2.2; serum lactate 1.5 mmol/L, normal < 2.0).

Brain MRI showed mild signal abnormalities in the dorsal periventricular white matter and increased signal intensities bilaterally on T2-weighted sequences in the nucleus caudatus and putamen, although brainstem and cerebellum were normal.

On examination at 14 years of age, she had short stature (height < 3rd percentile; weight < 3rd percentile). She had a slightly ataxic gait. Cognitive development was impaired — she could not read but was able to count up to 10.

#### 2.1.2 Patient 2

The younger sister of patient 1 had normal birth, and her motor development was slightly delayed (crawling at 9 months of age, walking at 22 months of age). There was a moderate delay in her speech development (2 word sentences at 3 years of age) with mild cognitive dysfunction. Diagnostic work-up took place at 5 years of age.

Her weight was < 10th percentile, and height < 3rd percentile. Cranial nerves were normal; she had no ptosis or ophthalmoparesis, had normal vision and hearing, but mild facial hypotonia. She had generalised muscular hypotonia but muscle strength and deep tendon reflexes were normal. There was no ataxia or dysmetria, however she had some intention tremor. She could walk and run, but could not ride a tricycle. Her speech was limited to short sentences and she had mild cognitive dysfunction.

She had asthma, mildly increased TSH with normal thyroid function, and heart, liver and gastrointestinal tract were normal. Because of her short stature, growth hormone therapy was considered.

Laboratory investigations showed normal results including metabolic workup, except for a moderately increased serum lactate (3.2 mmol/L, normal < 2.2). Brain MRI and MR spectroscopy at 4 years of age were normal.

### 2.2 Histological and biochemical analyses of skeletal muscle

Histological and biochemical analyses of skeletal muscle were performed at 6 years of age as previously described (Gempel et al., 2007).

### 2.3 Genetic analyses

Mitochondrial DNA deletions, depletion and point mutations were excluded in muscle DNA. Direct sequencing of *POLG*, *EFG1*, *EFTu*, *EFTs*, *MRPS16* and *TRMU* in blood-DNA of patient 1 was normal (Kemp et al., 2011).

#### 2.3.1 Exome sequencing

Exome sequencing was performed in genomic DNA of patient 1 by AROS Applied Biotechnology AS (Aarhus, Denmark) using Illumina's TruSeq DNA Sample Prep Kit and Exome Enrichment Kit, with 100 bp paired-end reads. Samples were processed on the Illumina HiSeq 2000 platform (Horvath et al., 2012). Sequence was aligned to the human reference genome (UCSC hg19) using BWA and reformatted using SAMtools. Single base variants were identified using VarScan (v2.2) and Indels were identified using Dindel (v1.01). The raw lists of variants were filtered using in-house Perl scripts to identify on-target variants that were rare with a minor allele frequency of less than 0.01 or not present in 1000 Genomes (Feb 2012 download), dbSNP135 or in the exome sequences of 91 unrelated and unaffected individuals. Putative ‘disease causing’ mutations were identified using MutationTaster (http://www.mutationtaster.org/). Primer sequences used to sequence *MTFMT* genomic DNA and cDNA are listed in the Supplementary materials.

### 2.4 Tissue culture and mitochondrial functional studies

Cultured myoblasts of patient 1 and controls were grown in skeletal muscle cell growth medium and supplement mix (PromoCell) plus 10% FBS (Gibco), 1% 200 mM l-glutamine (GIBCO) and gentamicin 50 μL/mL. Myoblasts of patient 1 and a control cell line were immortalised by transduction with a retroviral vector expressing the catalytic component of human telomerase (htert) (Lochmüller et al., 1999).

#### 2.4.1 Immunoblotting

SDS PAGE was performed on immortalised control and patient myoblasts. Aliquots of total protein (5–20 μg) were loaded on 14% sodium dodecyl sulphate polyacrylamide gels then transferred to polyvinylidene fluoride membranes. Membranes were subsequently probed with the following monoclonal antibodies: β actin, 0.1 μg/mL (SIGMA); complex IV subunit II, 1 μg/mL; complex II 70 kDA subunit, 0.1 μg/mL; and complex I NDUFB8 subunit, 0.5 μg/mL (MitoSciences). Following incubation with horseradish peroxidase-conjugated secondary antibodies (Dako, Denmark A/S) detected proteins were visualised by ECL-plus (GE Healthcare). Immunoblotting for MTFMT was performed on control and patient myoblasts. Aliquots of total protein (50–100 μg) were loaded on 14% sodium dodecyl sulphate polyacrylamide gels then transferred to polyvinylidene fluoride membranes. Membranes were subsequently probed with monoclonal antibodies for anti-MTFMT 2 μg/mL (Abcam) and complex II 70 kDA subunit, 0.1 μg/mL (Abcam), incubated with horseradish peroxidase-conjugated secondary antibodies (Dako, Denmark A/S) and detected proteins were visualised by ECL-plus (GE Healthcare).

#### 2.4.2 Blue native polyacrylamide gel electrophoresis (BN-PAGE)

BN-PAGE was performed on myoblasts of patient 1 and control myoblasts as previously described (Leary and Sasarman, 2009). After electrophoresis activities, “in gel” assays were carried out as previously described (Diaz et al., 2009). A Coomassie blue staining was done in parallel to the activities as a loading control.

#### 2.4.3 High resolution Northern blot analysis

Total RNA from 1 to 2 × 10^6^ cultured myoblast lines was extracted using TRIzol reagent (Life Technologies, Paisley, UK) according to the manufacturer's instructions. High resolution Northern blot analysis of total RNA (1 μg) was performed as previously described (Taylor et al., 2003). The human mt-tRNA^Met^ probe was generated using the forward primer H4404 (positions 4404–4426) 5′-TAAGGTCAGCTAAATAAGCTATC-3′ and reverse primer L4466 (positions 4466–4446) 5′-TACGGGAAGGGTATAACCAAC-3′. The human mt-tRNA^Glu^ probe was generated using the forward primer L14635 (positions14810–14791) 5′-TACTAAACCCACACTCAACAG-3′ and reverse primer H14810 (positions14810–14791) 5′-GGAGGTCGATGAATGAGTGG-3′. The radioactive signal for the mt-tRNA^Met^ probe was normalised to that of the 5S RNA probe for each sample.

## 3 Results

### 3.1 Histological and biochemical analyses of skeletal muscle

Muscle biopsy of patient 1 at 6 years of age detected mild lipid accumulation in type I fibres. There was a subsarcolemmal accumulation of mitochondria in type I fibres, but no typical ragged red fibres (RRF) on Gomori-trichrome staining, however oxidative enzyme staining for NADH NADH-CoQ-Oxidoreductase (NADH), succinate dehydrogenase (SDH) and COX showed more prominent mitochondrial networks.

Biochemical analysis of the RC enzymes in skeletal muscle of patient 1 showed a reduction of complex I (NADH-CoQ-Oxidoreductase (0.10 U/U citrate synthase (CS), normal range: 0.17–0.56 U/UCS) and COX (0.7 U/UCS, normal range: 0.9–4.7 U/UCS)). The activities of complex II, complex III and the pyruvate dehydrogenase were normal (CS was 100 U/gNCP, normal 45–100 U/gNCP). COX activity in fibroblasts of patient 1 was normal (0.91 U/UCS, normal 0.4–2.1 U/gNCP).

### 3.2 Genetic analyses

**Fig. 1:**
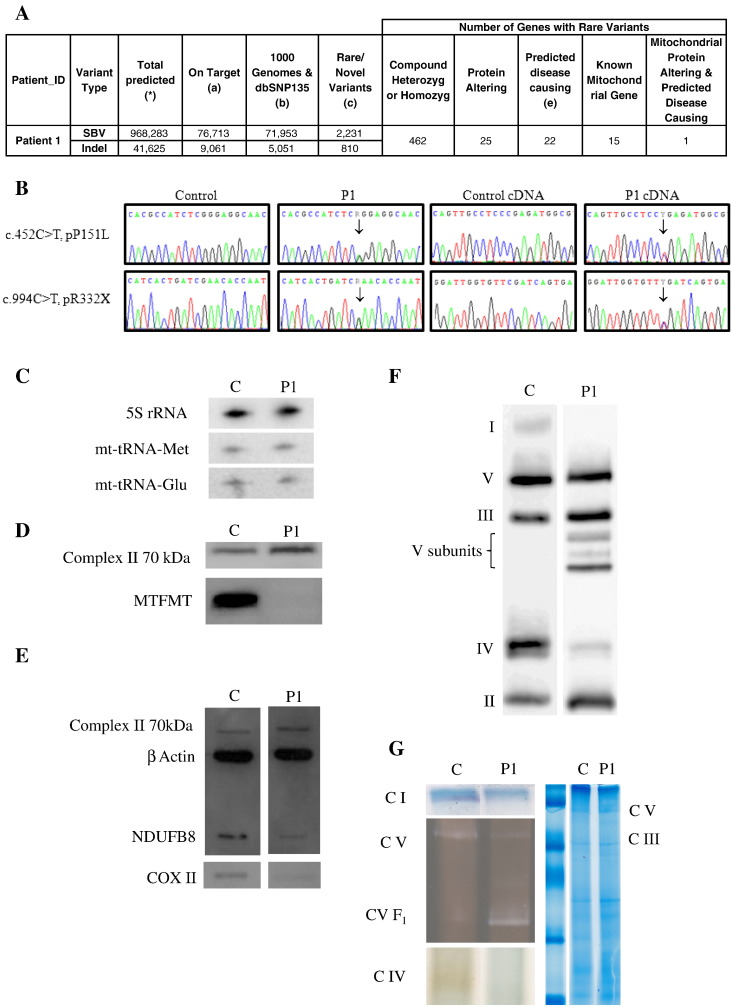
A) Variant numbers following analysis pipeline of BWA (Sequence Aligner); VarScan (single base variant — SBV calling); Dindel v1.01 (Indel calling). (*) SBV — VarScan parameters min. total coverage ≥ 5-fold, min. variant coverage ≥ 3-fold, min. Quality > 10; Indel — Dindel output filter min. variant coverage ≥ 4. (a) Variants with position within targets (Illumina TruSeq 62 Mb) +/− 500 bp, seen on both (forward & reverse) strands and (SBVs only) variant allele frequency > 24%; (b) variants that match 1000 genomes (Feb 2012) and/or dbSNP135 with minor allele frequency (MAF) > 0.01; (c) rare/novel variants (MAF < 0.01) with exclusion of common variants found to be shared in an in-house panel of 91 ʻnon-respiratory complexʼ individuals; (e) MutationTaster predictions. B) Identification of pathogenic compound heterozygous mutations in *MTFMT*. Electropherogram of *MTFMT* DNA and cDNA sequence of patient 1 and a control. C) High resolution Northern blotting detected normal mt-tRNA^Met^ steady state levels in myoblasts of patient 1. For comparison we show also mt-tRNA^Glu^ and 5S rRNA. D) SDS-PAGE immunoblotting analysis in myoblasts of patient 1 showed no detectable MTFMT protein. Complex II 70 kDa subunit antibody was used as a loading control. E) Immunoblotting in myoblasts of patient 1 showed decreased steady state levels for COX II and NDUFB8. Blotting with antibodies against the complex II 70 kDa subunit and β actin showed equal loading. F) BN-PAGE in myoblasts of patient 1 showed severely decreased complexes I and IV and 3 additional bands with complex V antibodies, however complex III was normal. G) In-gel activities of complexes I and IV were decreased in myoblasts of patient 1, and complex V was slightly reduced, but showed an extra band.

Exome sequencing identified 462 rare variants (Fig. 1A), which were not listed on dbSNP135 and were not found to be shared in the 1000 genome project or in a panel of 91 in-house disease control subjects (Horvath et al., 2012). Twenty-two of these variants were predicted to be disease-causing (MutationTaster), but only one gene, *MTFMT* contained two likely pathogenic variants (Fig. 1B). Only variants in *MTFMT*, a recently described human disease gene encoding a mitochondrial protein (Tucker et al., 2011) were confirmed on Sanger sequencing. Patient 1 was heterozygous for both a missense mutation, c.452C>T, p.P151L, and a nonsense mutation, c.994C>T, p.R332X in *MTFMT*. The mutations were not present in 91 in-house exomes, 128 Caucasian controls, or data from the 1000 genome project. The unaffected mother was heterozygous for the c.994C>T, pR332X variant and the affected sibling was compound heterozygous for both variants (Fig. 1B). DNA was not available from the father. Sequencing of *MTFMT* cDNA in patient 1 showed both changes to be present at transcript level (Fig. 1B), suggesting that the stop mutation in the last exon does not result in nonsense mediated decay. Direct sequencing of the *MTFMT* gene in 30 patients with combined RC deficiency detected no pathogenic mutations.

### 3.3 Tissue culture and mitochondrial functional studies

High resolution Northern blotting showed normal steady state level of mt-tRNA^Met^ (Fig. 1C). Immunoblotting for MTFMT detected no band in myoblasts of patient 1, confirming a severe decrease of the protein (Fig. 1D). COXII and NDUFB8 were also decreased in patient 1, suggesting a deficiency in complexes I and IV respectively (Fig. 1E). This was confirmed by BN-PAGE which showed an absence of complex I and severe decrease in complex IV, but also detected a number of extra bands with the complex V antibody suggesting an unstable ATP synthase (Fig. 1F). Complex III was normal. In-gel activities were corresponding to the BN-PAGE result (Fig. 1G).

## 4 Discussion

Pathogenic mutations in the *MTFMT* gene have been reported in 2 independent patients with combined RC defect (Tucker et al., 2011) and 2 with isolated complex I deficiency to date (Haack et al., 2012) (Table 1). All reported patients had Leigh syndrome, however the clinical presentation was relatively late-onset in 3 patients, 2 of them being ambulatory in adult age. The disease progression was subtle through the second decade, although acute metabolic crisis and respiratory arrest lead to death in one patient after short anaesthesia for MRI (Tucker et al., 2011). The very brief description of 2 further patients (Haack et al., 2012) did not allow us to make assumption on the clinical severity.

Here we describe 2 sisters with compound heterozygous mutations in the *MTFMT* gene leading to combined RC deficiency. Similarly to the patients reported by Tucker et al., both sisters had normal development in their early years, the first clinical investigations took place at 9 and 5 years of age. The first symptoms were an impaired speech and mild cognitive delay, while motor symptoms and ataxia were subtle and became obvious in a later stage of the disease. Brain MRI detected signs typical for Leigh syndrome in patient 1, but was normal in patient 2 at 4 years of age, suggesting that MRI may not be positive in the early phase of the disease.

One of the mutations identified in our patients, the nonsense mutation c.994C>T, p.R332X has been previously reported in compound heterozygous state with another nonsense mutation (c.626C>T/p.Arg181SerfsX5) (Haack et al., 2012). The second mutation c.452C>T, p.Pro151Leu is novel, leads to the exchange of a well conserved amino acid and is predicted to be disease causing (MutationTaster). It has not been detected in the international SNP databases and in the 1000 genome database, and was absent in 256 ethnically matched normal Caucasian control chromosomes. The clinical presentation co-segregated with the compound heterozygous mutations within the family. The pathogenicity was verified by the lack of detectable MTFMT protein on immunoblotting (myoblasts of patient 1). Mitochondrial proteins showed decreased steady state levels in myoblasts of patient 1 on immunoblotting and BN-PAGE detected a severely abnormal pattern with decreased complexes I and IV and several abnormal smaller bands stained with complex V antibodies. These findings were keeping with the combined RC defect in skeletal muscle. High resolution Northern blotting showed normal steady state of mt-tRNA^Met^, suggesting that the defect in formylation does not affect mt-RNA^Met^ stability.

How can we explain the combined RC defect in MTFMT deficiency? In metazoan mitochondria, after aminoacylation of tRNA^Met^ has occurred, Met-tRNA^Met^ is used for both translation initiation and elongation. This is unlike its bacterial counterpart whose translational machinery contains individual tRNA^Met^ molecules for each role (Tucker et al., 2011). In order to initiate translation in humans, a proportion of Met-tRNA^Met^ is formylated by mitochondrial methionyl-tRNA formyltransferase (MTFMT) producing fMet-tRNA^Met^. The translation initiation factor (IF-2mt) has a high affinity for fMet-tRNA^Met^ and promotes its binding to the mitochondrial ribosome (Spencer and Spremulli, 2004). However, if Met-tRNA^Met^ binds to EFTu before it is formylated, it acts as an elongator tRNA. This partitioning mechanism requires that IF-2_mt_ strongly discriminates against Met-tRNA^Met^ and that EF-Tu_mt_ preferentially binds Met-tRNA^Met^ (Spencer and Spremulli, 2004). Based on this hypothesis a defect in Met-tRNA^Met^ formylation would affect mitochondrial translation, although ^35^S methionine labelling in our patient showed only mild abnormalities (patient 12 in Kemp et al., 2011). This result is in contrast with the severe impairment on BN-PAGE, suggesting that there is still more to discover about the disease mechanism.

## 5 Conclusions

Mutations in *MTFMT* should be screened in patients with Leigh syndrome and combined respiratory chain deficiency. Exome sequencing is a very powerful diagnostic tool, and its value in clinical medicine is enormous, however it can only be optimally exploited if combined with detailed phenotyping. The selection of primary disease causing changes can be facilitated by functional studies.

## Figures and Tables

**Table 1:** Summary of all previously reported patients compared to our patients with *MTFMT* mutations.

Ref.	Onset	Age/death	Clinical presentation	Brain MRI	Muscle biopsy	Fibroblasts RC	Mutation
*Previously described patients*							
Tucker	9 years	Alive at 21 years	Optic atrophy, ophthalmoparesis, speech problems, ataxia, cognitive impairment, WPW syndrome	LS	Complexes I+IV↓	Fibroblasts Complexes I+III+IV↓	c.626C>T p.Arg181SerfsX5 c.382C>T/p.Arg128X Compound heterozygous
	9 years	Alive at 18 years	Global developmental delay, optic atrophy, speech problems, ataxia, cognitive impairment, WPW syndrome	LS	n.d.	n.d.	
Tucker	5 years	Died at 5 years	Weight gain, hypertension, Cushing's disease, after general anaesthetics seizure, respiratory failure	LS	Complexes I+III+IV↓	Fibroblasts Complexes I+III+IV↓	c.626C>T p.Arg181SerfsX5 c.374C>T/p.Ser125Leu Compound heterozygous
Haack	n.d.	n.d.	Vertical gaze palsy, optic atrophy, tetraspasticity, mental retardation, bladder dysfunction	LS	Complex I↓ 16% of normal	n.d.	c.626C>T p.Arg181SerfsX5 c.994C>T/p.Arg332X Compound heterozygous
Haack	n.d.	n.d.	Developmental delay, muscular hypotonia, ataxia	LS+white matter lesion	Complex I↓ 12% of normal	n.d.	c.626C>T p.Arg181SerfsX5 Homozygous

*Patients described in this paper*							
P1	3 years	Alive at 16 years	Developmental delay (more speech than motor), muscular hypotonia, ataxic gait, dysarthria	LS	Histology mt accumulation complexes I+IV↓	Myoblast Complexes I+IV↓	c.452C>T, p.Pro151Leu c.994C>T, pR332X Compound heterozygous
P2	5 years	Alive at 6 years	Developmental delay (more speech than motor), muscular hypotonia, tremor	Normal	n.d.	n.d.	

Abbreviations: +: present, n.d.: not determined; LS: Leigh syndrome, RC: respiratory chain.
