# Supplementary material for: Clinical and functional characterisation of the combined respiratory chain defect in two sisters due to autosomal recessive mutations in MTFMT
Source: Mitochondrion. 2013 Nov;13(6):743–8. doi: 10.1016/j.mito.2013.03.002 (PMC4046648; doi:10.1016/j.mito.2013.03.002)
Supplement: Supplementary file 1 — Supplementary material [file mmc1.docx]

**Supplementary materials**

*MTFMT* intronic primer sequences for direct sequencing in genomic DNA

| MTFMT | Forward Primer | Reverse Primer |
| --- | --- | --- |
| Exon 1 | TCACCAACTCCAACCAGACC | AGAAGTCCAAAACCCTCGGG |
| Exon 2 | tgcttcatgagattaatcataaacat | cagaaagcatagagttcaaattatca |
| Exon 3 | TTCCTTTCAGGTGATACGAGAC | GAGACCCCGCATAAATACCA |
| Exon 4 | cagcctcctgagcaactagg | gagatcacaaatgccaggcta |
| Exon 5 | tgaggtttccccaagtagaca | cacacaaaagtgggcactca |
| Exon 6 | ttgagaggttagaatgaattctctg | tcatttctttcccctcacct |
| Exon 7 | cggtctgttccatcaaactt | tgagaatgagtgttgcaaagaa |
| Exon 8 | gcaaatgtgttctctccggta | atgccttggcaaaacttcat |
| Exon 9 | gtcaaaaaccgcaatgactttt | cagataattccttgtaaa |

Primer sequences and annelaing temperatures for sequencing of *MTFMT* cDNA in 3 overlapping fragments

| *MTFMT* cDNA | Forward Primer | Reverse Primer | Annealing Temp °C |
| --- | --- | --- | --- |
|  |  |  |  |
| Fragment 1 | gcgatgagggtgttggtgTGTAAAACGACGGCCAGT | ccgtgaagcactgtatggatt | 65 |
|  |  |  |  |
| Fragment 2 | aggactgccagtgaagcaat | ccaatggcacggtaaagtct | 65 |
|  |  |  |  |
| Fragment 3 | gtgccaacatgctcatttca | ggtttttatccatcttcttcctaaCAGGAAACAGCTATGACC | 65 |
|  |  |  |  |
